# Effect of Microwave Power and Gas Flow Rate on the Combustion Characteristics of the ADN-based Liquid Propellant

**DOI:** 10.3390/ma16010147

**Published:** 2022-12-23

**Authors:** Sheng Pan, Chenghao Zhao, Dechao Zhang, Yangyang Hou, Gaoshi Su, Xuhui Liu, Yusong Yu, Jiannan Shen

**Affiliations:** 1 Hydrogen Energy and Space Propulsion Laboratory, School of Mechanical, Electronic and Control Engineering, Beijing Jiaotong University, Beijing 100044, China; 2 Beijing Institute of Control Engineering, Beijing 100190, China; 3 China Nuclear Power Engineering Co., Ltd., Beijing 100840, China

**Keywords:** ammonium dinitramide, microwave ignition, plasma torch, combustion characteristics

## Abstract

As a new type of energy-containing material, Ammonium dinitramide based liquid propellant has the advantages of being green, having low toxicity, good stability, and high safety performance. Traditional catalytic combustion methods require preheating of the catalytic bed and deactivation of the catalytic particles at high temperatures, while microwave ignition methods can effectively solve these problems. To study the combustion characteristics of ADN-based liquid propellants during microwave ignition, the influence of microwave power and gas flow rates on the combustion process are analyzed using experimental methods. A high-speed camera was used to observe the enhanced effects of microwave power and gas flow on plasma and flame. Combined with temperature measurement, the combustion process of ADN-based liquid propellants under the action of plasma was analyzed. The combustion process in the presence of microwaves was observed by comparing parameters such as flame length, flame temperature, and radical intensity. Those results show that, with the increase in microwave power, the luminous burning area of the flame grows significantly. The microwave power is increased by 250 W each, and the flame jet length is increased by nearly 20%. The increase in microwave power also leads to an increase in propellant combustion temperature, however, this increase gradually slows down. At a gas flow rate of 20 L/min, the ADN-based liquid propellant showed the best combustion performance with a maximum jet length of 14.51 cm and an average jet length increase of approximately 85.9% compared to 14 L/min. Too much gas flow rate will hinder the development of the jet, while the high-velocity airflow will have a cooling effect on the flame temperature. The results provide a basis for the specific parameter design of microwave ignition and promote the application of ADN-based liquid propellants in the aerospace field.

## 1. Introduction

Launching a spacecraft is an economically demanding task that can lead to environmental pollution and even safety accidents. As the competition for outer space resources among the world’s major spacefaring nations becomes more intense, the need to find a new type of safe [1,2], reliable, green, and economical propellant has become a key development goal for all countries. Ammonium dinitramide (ADN) based liquid propellant, a green energy-containing material that is safer, less polluting, and less costly than traditional hydrazine propellants, is gradually receiving attention from countries around the world [3].

The Swedish Space Corporation (SSC) first tried to apply ADN-based liquid propellant to the main star of the PRISMA dual-technology experimental satellite “Mango” developed by ECAPS. Russia, Germany, Japan, and other countries have carried out corresponding experiments and studies on ADN [4,5,6,7,8,9,10]. Talawar et al. [11] from India, proposed to Russia, the idea of using ADN for artillery propellants and rocket propellants. Yuichiro et al. [12] found that by adjusting the unit components of ADN, it was able to achieve higher specific impulse of ADN-based liquid propellants than conventional hydrazine propellants, as well as advantages such as better ignition-rich characteristics and easy storage due to low freezing point. Piyush et al. [13] performed gas-phase coupled kinetic simulations of ADN monopropellant combustion and found that the combustion rate and surface temperature of ADN were in the interval from 0.7 to 350 atm, removing the interval from 60 to 100 atm, during which it showed an increase in combustion rate with increasing surface pressure. Yuichiro et al. [12] presented the safety elements of low-temperature storage and transportation of ADN-based liquid propellants. Li et al. [2] carried out hot spot ignition and combustion experiments in different oxidizing atmospheres. They carried out ignition and combustion experiments in oxidizing gas atmospheres such as air, O_2_, N_2_O, and NO, analyzing the ignition delay time, combustion process, and changes of ADN droplets during the period. The experiments conducted have shown that ADN-based liquid propellants have the advantages of fast ignition, high energy, adequate combustion, clean emission, environmental friendliness, and easy storage and transportation. The current ignition method of ADN-based thrusters mostly uses catalytic ignition and has been successfully applied in satellite propulsion systems. Researchers have carried out a large number of experimental and numerical simulation studies on the catalytic combustion process. Yu et al. [14] numerically studied the evolution of decomposition and combustion in ADN-based thrusters and systematically investigated the effect of structural parameters of the catalyst bed (length, diameter, and wall thickness) on the overall performance of ADN-based thrusters, and found that as each of the three structural parameters rises, the temperature will first increase then decreases, and there exists the optimal design value that makes the highest temperature. By comparing the maximum temperature at the exit of the combustion chamber with the specific impulse, it can be obtained that the wall thickness has an important role in the overall performance of the ADN-based thruster, while the length of the catalyst bed has a weak effect on the general performance among the three structural parameters. Zhang et al. [15] numerically investigated the catalytic decomposition and combustion characteristics of ADN-based thrusters from the perspectives of composition distribution, temperature, and pressure, and showed that the porosity and preheating temperature of the catalytic bed had significant effects on the decomposition and combustion characteristics, and the thrust performance improved with the increase in the porosity and preheating temperature of the catalytic post-bed. Maleix et al. [16] found that ADN catalytic beds require catalyst carrier materials that can withstand higher temperatures (approximately 1650 °C and 1900 °C) and exhibit sufficient porosity and resistance to sintering and selected novel catalytic bed materials for their study.

The application of energy-containing ionic materials (ADN) has been of interest to scholars, and microwave ignition methods have been proposed for use in the field of space propulsion for their advantages of stable ignition and high ignition energy. To conclude, since the catalytic ignition method has high requirements on the material of the catalytic bed, catalyst performance, preheating temperature, etc., and all the above factors will have an impact on the catalytic decomposition and ignition combustion of ADN, research teams in various countries are also trying to find a new ignition method, for example, ignition using the electric spark plug proposed by Wilhelm et al. [17], Michele et al. [18] proposed ignition using a torch igniter, and electrolytic decomposition ignition for MEMS cold gas thrusters [19], microwave ignition, etc. have received extensive attention, and this paper will focus on further research on microwave ignition. The mechanisms of microwave plasma ignition is mainly “high-temperature effect”, “chemical effect” and “jet effect” [20,21]. The use of microwave-assisted ignition technology in automotive internal combustion engines is a very mature industry. Plasma has a significant combustion-enhancing effect on energy-containing materials, high-energy particles will be hydrocarbon compounds in the carbon-carbon bonds and hydrocarbon bonds and other chemical bond destruction so that large molecules of hydrocarbons turn into small molecules of hydrocarbons to improve chemical activity. A large number of experiments have been carried out to obtain positive progress and results in this field. Ikeda et al. [22] tried a microwave plasma-assisted ignition system for a single-cylinder internal combustion engine and ignited a thin methane-air mixture and found that microwave plasma-assisted ignition was also stable in the thin state. Hwang et al. [23] conducted microwave plasma-assisted ignition experiments using an acetylene-air mixture in a 1.4 L constant volume combustion chamber to observe the effect of microwaves on flame development and finally found that the flame speed increased by 20% with microwave plasma-assisted ignition, and that microwave ignition had a better combustion index than conventional spark plug ignition.

In this paper, a microwave ignition system adapted to ADN-based thrusters is established to promote the combustion of ADN-based liquid propellants through microwave ignition of air plasma, in order to study the influence of microwave power and gas flow rate on the combustion characteristics of the plasma jet and ADN-based liquid propellants, and to explore the optimal ignition parameters and ignition scheme for achieving ADN-based liquid propellants.

## 2. Materials and Methods

### 2.1. Materials

**Figure 1:**
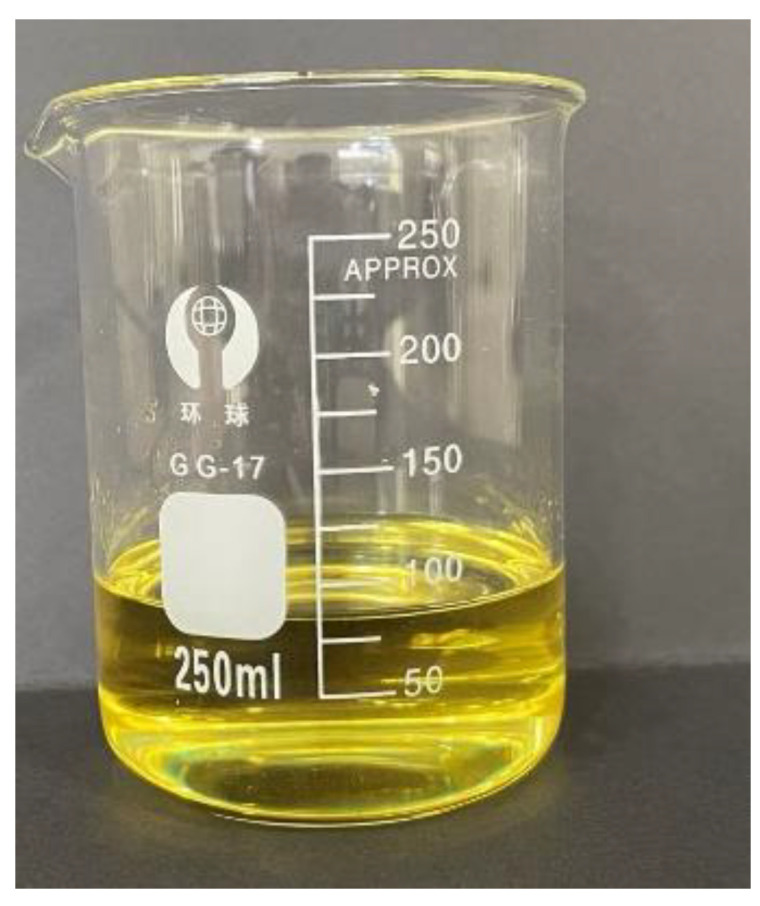
ADN-based liquid propellants at ambient temperature and pressure (A high-energy ionic liquid propellant).

Ammonium dinitramide (ADN) is a high-energy-density material, generally a white or light yellow solid at ambient temperature and pressure, consisting of an amino cation (NH_4_^+^) and a dinitramide anion (N(NO_2_)_2_^−^). Researchers have developed different series of ADN-based liquid propellant formulations and conducted performance tests by configuring ADN, water, and fuel into aqueous solutions in a certain ratio, taking advantage of ADN’s solubility in water. Figure 1 shows the ADN-based liquid propellant that we used in experiments.

### 2.2. Design of the Resonant Cavity

Microwave ignition devices use the frequency of microwaves to generate a fast-oscillating electric field, which generates a strong electric field in a specific device, thereby prompting gas discharge to form plasma. BJ 26 type waveguide (NEWSAIL, Zhengzhou, China) is selected for this experimental device, and the metal waveguide can only propagate transverse electric (TE) and transverse magnetic (TM) waves. After calculation, the cut-off frequencies of the two waves are 3.90 GHz and 1.79 GHz, and the microwave input frequency of this experiment is 2.45 GHz, so the main mode of waveguide transmission in this experiment is the TE10 mode. However, the BJ26 type waveguide needs high incident power to break down the air, so the BJ 26 type waveguide is modified and designed in this experiment. The internal dimension of the standard BJ 26 waveguide is a rectangle, the length of the wide side is a = 86 mm, and the length of the narrow side is b = 43 mm. The transmission power of the rectangular waveguide is

(1) P=12Re∫0a∫0b−EyHx*dxdy=ab4E2Z

where *E* is the electric field strength and *Z* is the equivalent impedance of the waveguide.

Since the TE10 mode wave impedance is only related to the broad side a and has nothing to do with the narrow side b, when the transmission power remains unchanged, the narrow side of the waveguide is generally reduced to 1/2 of the original length to enhance the cavity. Electric field strength, the transmission power after narrow-side reduction can be obtained as

The transmission power before narrow-side reduction is

(2) $$P_{1}=\frac{E^{2}ab}{4Z_{{\mathrm{TE}}_{10}}}$$

The transmission power after narrow-side reduction is

(3) $$P_{2}=\frac{E^{’2}ab}{8Z_{{\mathrm{TE}}_{10}}}$$

When the transmission power remains unchanged, that is, $P_{1}=P_{2}$, and formulas (2) and (3) are combined, when the narrow side is reduced to 1/2 of the original length, the electric field intensity in the cavity is increased by 1.44 times.

**Figure 2:**
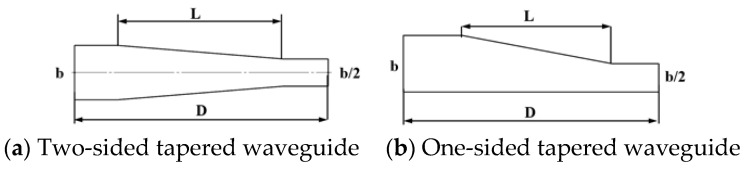
Structures of two reduced waveguides.

There are two design methods for shortening the narrow side of the waveguide: (a) reducing both sides at the same time; (b) reducing only one side, the schematic diagram is shown in Figure 2. To determine the specific value L of the reduction length of the resonator, the simulation software Comsol Multiphysics (Version 5.6) is used to simulate the model. Under the input frequency of 2.45 GHz, the BJ 26 rectangular waveguide has a waveguide wavelength of λg = 173.6 mm in the TE10 mode. According to Poynting’s theorem, when the cavity length is 3 λg/2, a higher electric field intensity can be obtained in the cavity, so the total length D of the device is set to 260 mm.

The mathematical model of the simulation is based on the electromagnetic field theory of the Maxwell equation, and the Helmholtz equation is derived by combining the constitutive relation. In a steady state analysis, the Helmholtz equation is:

(4) $$\nabla \times \left(\mu^{-1}\nabla \times E\right)-k_{0}^{2}\left(\varepsilon_{r}-\frac{j\sigma }{\omega \varepsilon_{0}}\right)E=0$$

where is the permittivity of the dielectric, *σ* is the conductivity, *μ* is the permeability, and *k* is the wavenumber of the dielectric.

**Figure 3:**
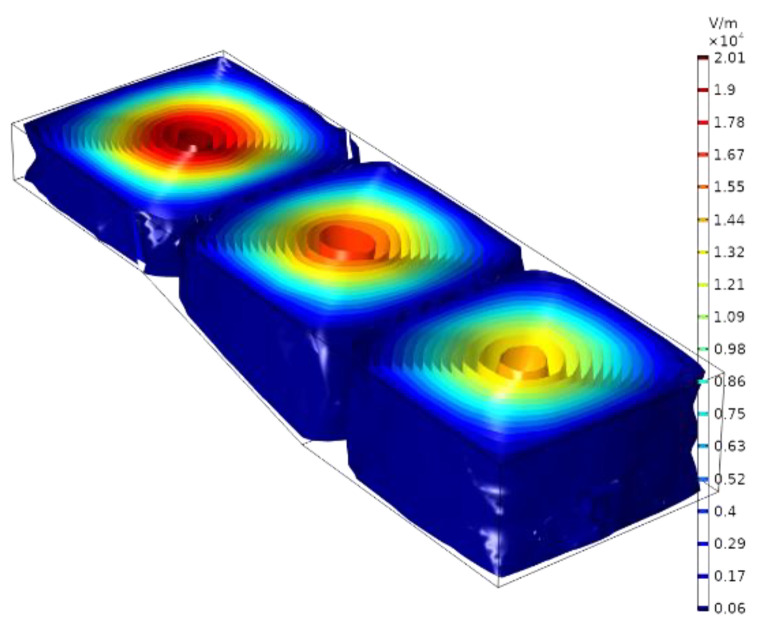
Electric field intensity distribution in the resonator.

As shown in Figure 3, when the microwave power is 100 W, the strongest electric field exists at 1/4 of the resonance wavelength. According to the electric field distribution in the cavity, the position of the opening for placing the quartz tube is set at 1/4 resonance wavelength away from the short-circuit end face. Two openings are, respectively, designed at the upper and lower ends at 1/4 of the resonance wavelength from the resonant cavity, for inserting the quartz tube and the subsequent input of gas and ADN-based liquid propellant. Because the larger the aperture diameter, the more obvious the weakening of the electric field; if the size is too small, the processing difficulty increases sharply. Therefore, under the premise of satisfying the input of air intake and propellant flow, the aperture should be reduced as much as possible to improve the utilization rate of microwaves. The final selected aperture is 28 mm. The inner diameter of the matching quartz tube is 25 mm, the outer diameter is 28 mm, and the height of the quartz tube is 15 cm.

### 2.3. Experimental System

**Figure 4:**
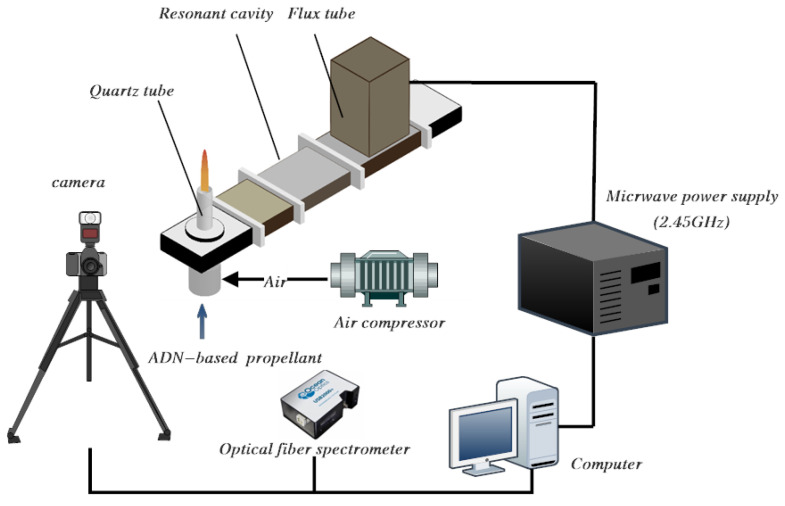
Schematic diagram of microwave plasma ignition experimental platform.

Figure 4 is a schematic diagram of a microwave plasma ignition experiment system, which is composed of a microwave generation component, a microwave plasma ignition component, and an experimental data acquisition system. The function of the microwave-generating component is to generate microwaves and transport them into the resonant cavity. The microwave generation component includes a microwave power supply, a magnetron, a circulator, a detector, a cooling water tank (including a water pump), and a waveguide connected to a microwave ignition component. The microwave power supply and the magnetron together constitute the microwave source. Circulators and detectors are used to control the direction of microwave transmission and monitor the reflected microwave power. The function of the water pump is to provide cooling water for the whole device, and the water in the load tank is used to absorb the reflected power. The function of the microwave plasma ignition component is to realize the coupling of microwaves in the cavity and decompose the gas to achieve ignition and combustion. The microwave ignition component is centered on a resonant cavity and includes a supporting connection device, a 550-9 air compressor (Fengbao, Shanghai, China), and a BT300-2J peristaltic pump (Longer, Shanghai, China). The main body of the microwave ignition component is a gradient reduction altitude resonant cavity based on the BJ26 waveguide. It is opened at a distance of 1/4 resonance wavelength from the short-circuit end and a quartz tube is placed through it. At the lower end of the resonant cavity, an aluminum connection device is assembled with the cavity to connect the peristaltic pump to the air compressor. Two connectors capable of connecting the trachea are set on the side of the connection device. The gas working medium is passed from both ends at the same time during the test to ensure the uniformity of the gas input and avoid the interference of the uneven gas distribution on the test on one side. The bottom end of the connection device is used as the propellant input end, and the propellant supplied by the peristaltic pump is nebulized and pumped into the combustion reaction area in the cavity by inserting the nozzle. The inner conductor is set in the fixed position of the tube and is used to couple the microwave energy to generate a strong electric field, penetrate the gas working medium, and stimulate the generation of microwave plasma. The gas working medium is sent into the resonant chamber through the self-connected device of the air compressor. The microwave energy is directly coupled into the designed resonance cavity through the BJ26 aluminum waveguide with ionized gas working medium in the quartz tube to generate an air plasma. The ADN-based liquid propellant is pumped into the resonant chamber through the opening of the lower end of the connection device of the peristaltic pump through a joint with a diameter of 0.2 mm and reacted with the microwave. The function of the experimental data acquisition system is to collect and record the jet height, flame temperature, and combustion spectrum of the flame in the experiment. It is mainly composed of a temperature sensor, a fiber optic spectrometer, and a high-speed camera. The temperature sensor uses a K-type armored thermocouple with a diameter of 2 mm and a length of 15 cm. Its maximum temperature resistance is 1573 K, and it can carry out long-term high-temperature measurements. The measurement error is 2%. It is fixed above the quartz tube by a clamping device 10 mm. The maximum spectrum measurement range of Ocean USB 2000+ fiber optic spectrometer (Ocean Insight, Orlando, FL, USA) is 200–900 nm, the resolution is 1.5 nm, and the detection integration time is 1 ms. In order to ensure that the measurement signals all come from the measurement target object, a collimating lens is added to the fiber optic probe. The high-speed camera model is Pho-Tron Nova S9 (Photron, San Diego, CA, USA). The maximum shooting speed of the camera is 193 frames per second (corresponding resolution: 256 × 128). In this experiment, under 1024 × 1024 (1 million) pixels, the acquisition frequency of the camera is set to 500 frames per second, which can better understand the instantaneous changes in the flame combustion process. In the experiment, a high-speed camera is used to photograph the changes in the flame during the combustion process, and to record the formation, development, and evolution of the flame in a stable combustion state.

### 2.4. Error and Uncertainty Analysis

In this experiment, through repeated experiments and measurements, the median value and the average value of the experimental data are taken to reduce random errors. Since the parameters in this experiment are directly measured, such as the temperature and length of the flame, the measurement error can be measured by the measuring instrument. The accuracy is obtained directly. For directly measured parameters, the uncertainty is expressed as:

(5) $$x_{i}=x_{i,means}\pm \delta_{x}$$

The known diameter of the quartz tube is D = 28 mm, $w_{D}$ = 1 mm; tube length l = 15 cm, $w_{l}$ = 5 mm; then the relative uncertainties of the tube diameter and tube length are:

(6) $$\frac{w_{D}}{D}=3.57\%,\frac{w_{l}}{l}=3.3\%$$

The measurement accuracy of the thermocouple used to measure the temperature is ±2% at high temperature. Combined with the maximum measurement range of 1573 K in the experiment, the maximum relative error range of the temperature can be obtained as 31.5 K, and the minimum jet temperature measured in the experiment is 567.9 K, then the maximum relative uncertainty of temperature is:

(7) $$\frac{w_{T}}{T}=5.55\%$$

The measuring instrument used for the gas flow rate is a rotameter, which is calibrated with air, and its accuracy is 4%, that is, the relative uncertainty of the airflow is 4%. The peristaltic pump is calibrated with pure water, and its accuracy is 1%, that is, the relative uncertainty of the propellant flow is 1%. The Table 1 is a summary of the Uncertainty of parameters.

## 3. Results and Analysis

### 3.1. Effect of Microwave Power on the Combustion Characteristics of Plasma Torch

**Figure 5:**
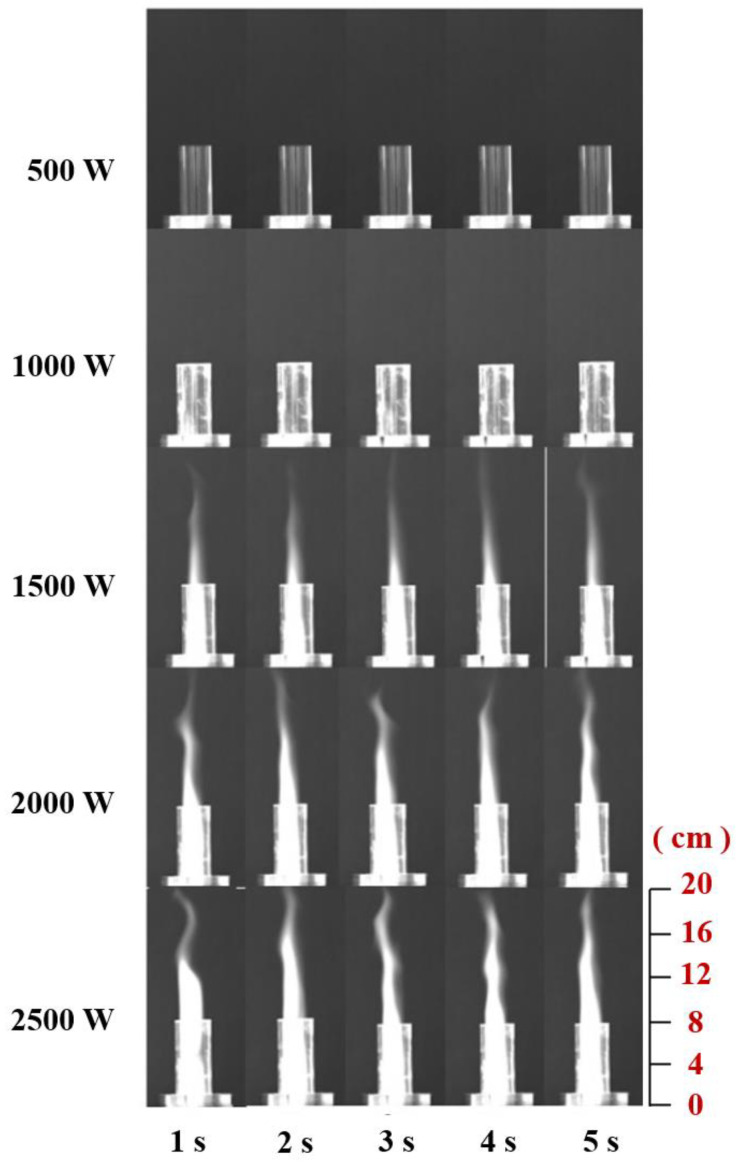
Plasma jets at different times with different microwave powers.

During the generation and maintenance of the plasma, energy is provided by the incoming microwaves and the microwave power directly influences the effect of the gas discharge in the resonant cavity. To ensure that the microwave power was a single variable factor in the experiment, a flow meter was used to stabilize the gas flow rate at 20 L/min before switching on the microwave generator assembly. The effects of microwave power on the generation of air plasma and the jet length were investigated experimentally when the microwave powers were 500 W, 1000 W, 1500 W, 2000 W, and 2500 W, respectively. Figure 5 shows the plasma jet images of burning 1–5 s under different microwave power. When the microwave power is 1000 W, a bright light is produced in the resonant cavity due to the gas discharge generating air plasma, but the length of the resulting plasma jet is small due to the low microwave power and the electric field strength only allows a limited amount of air to be ionized into plasma. As the microwave power was increased to 1500 W, the intensity of the gas discharge increased and the height of the plasma jet increased significantly, with the plasma jet already extending out of the resonant cavity and appearing inside the quartz tube. Continuing to increase the microwave power, the length of the plasma jet grows. When the microwave power is 2000 W and 2500 W, the plasma jet grows more in area, while the jet length growth is no longer as pronounced as in the previous period. The jet itself can be seen to fluctuate up and down for different times at the same power.

**Figure 6:**
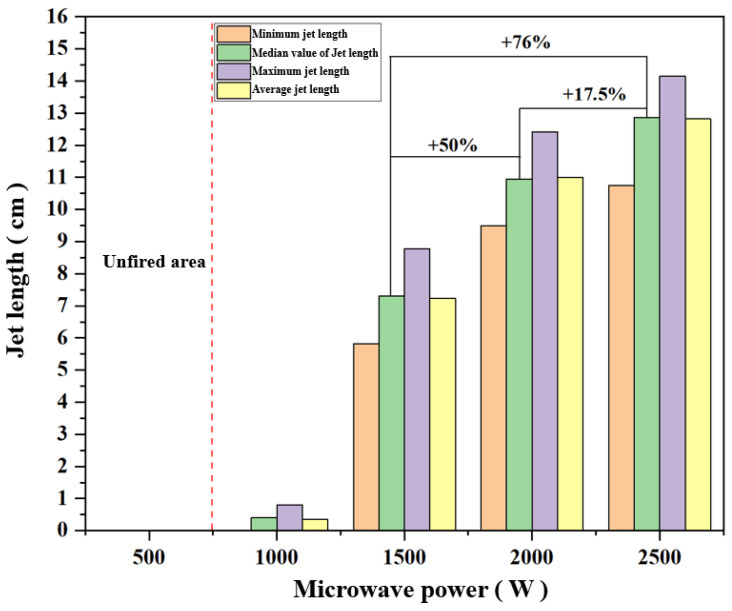
The median, average, and maximum value of jet length under different microwave power.

Figure 6 shows the median, mean and maximum values of the air plasma jet length at different microwave powers. The measurement of jet length is based on the contact surface between the quartz tube and the boss. The median values of plasma jets with different microwave powers in the experiment are 0 cm, 0.4 cm, 7.3 cm, 10.95 cm, and 12.87 cm, respectively. The difference between the average value and the median value of each power jet length above 1000 W is within 1%. When the microwave power was increased from 1500 W to 2000 W, the median value of the jet length increased by 50%, and when the microwave power was increased from 1500 W to 2500 W, the jet length increased by 76%, which means that the increase of the microwave power on the air plasma jet length is nonlinear, and the rate of this growth is gradually slowing down. The maximum value of the air plasma jet length at 1500 W power does not exceed the minimum value of the plasma jet length at 2000 W power, while the average value at 2000 W is already greater than the minimum at 2500 W. This phenomenon also explains the increase from the side. The increasing trend of microwave power to the plasma jet length is gradually weakening. In general, when the gas flow rate is fixed, the microwave power can increase the length of the plasma jet, but this gain is gradually weakened with the increase of the power. It can be guessed that when the power increases to a certain level, the gas will be ionized to the greatest extent to generate plasma so that the subsequent power increase cannot further increase the total amount of plasma, and the length and area of the jet will not increase.

**Figure 7:**
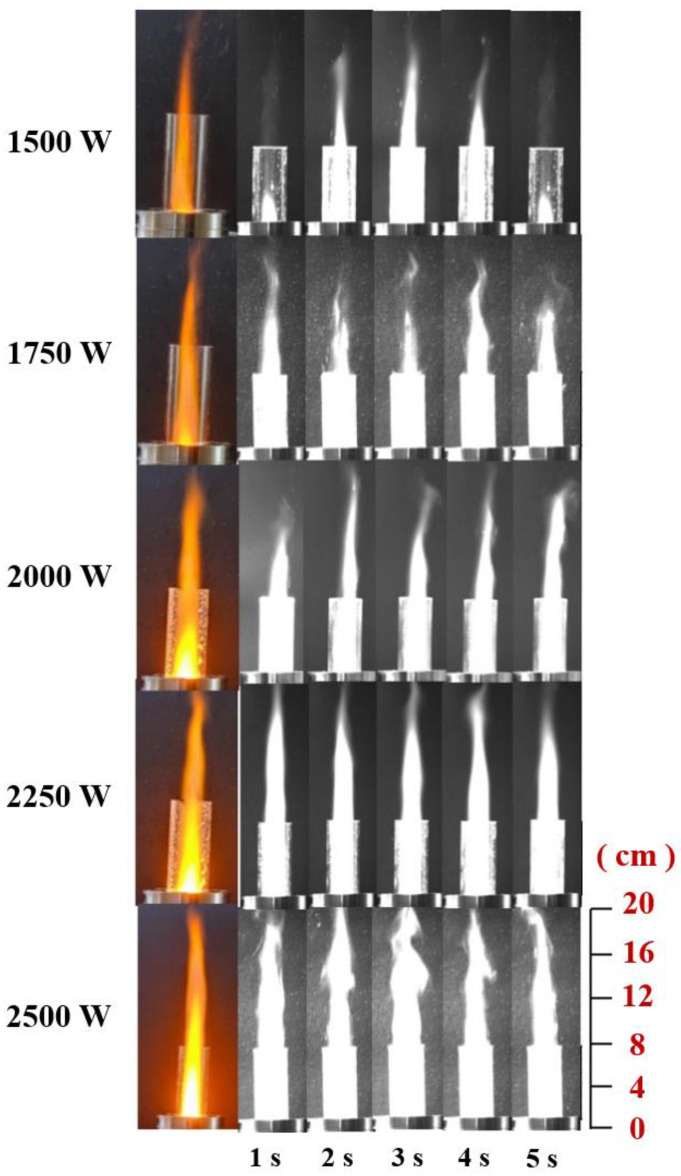
Combustion images of propellant at different times under different microwave power.

In the combustion jet experiment of ADN-based liquid propellant with microwave power, according to the experience obtained from the previous microwave power ionization experiment of the gaseous working medium, and the guidance of the minimum power of propellant ignition, keep the propellant flow rate at 20 mL/min, the gas flow rate was 20 L/min, and the microwave initial condition was increased from 500 W to 1500 W, and the combustion of ADN-based liquid propellant was tested under the action of five microwave energies: 1500 W, 1750 W, 2000 W, 2250 W, and 2500 W. Figure 7 shows the combustion images of ADN-based liquid propellants at different times under different microwave powers.

**Figure 8:**
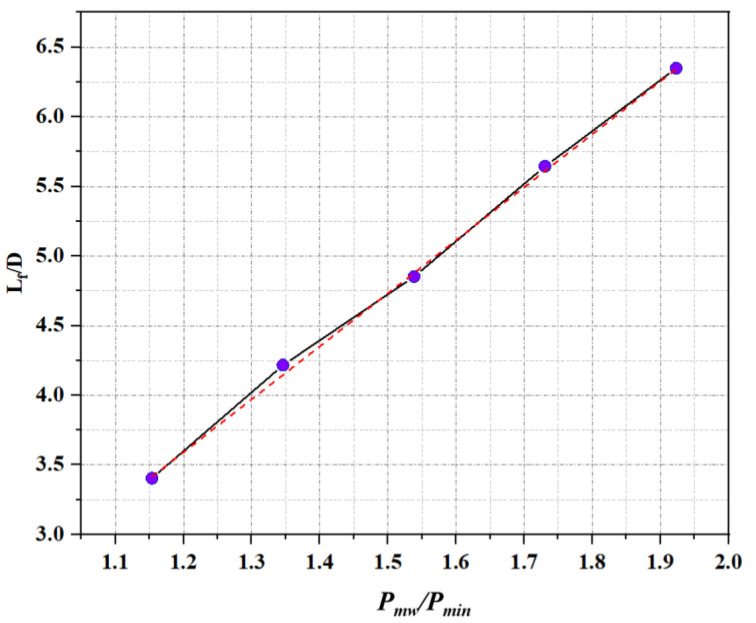
Dimensionless processing of flame length.

The input microwave power was compared to the lowest microwave power to achieve the ignition of ADN-based liquid propellant, and the median value of the jet length was compared with the inner diameter of the quartz tube to achieve dimensionless parameters. Figure 8 is the dimensionless processing of the flame jet length under the action of microwave power. Through the parameter fitting after dimensionless, it can be found that all data are distributed on both sides of a straight line, which is linearly distributed, and the straight line can be expressed as:

$$\frac{L_{f}}{D}=3.8\times P_{mw}∕P_{min}-0.96\left(R^{2}=0.98\right)$$

The microwave power has an enhancement effect on the jet length of the flame, and the limit of this enhancement effect is related to the combustion medium itself. When only the gas working medium was continuously fed in the experiment, although the length of the plasma jet generated by the microwave ionized gas working medium was still increased by the microwave energy, the increase ratio decreased from 2000 W to 2500 W. After adding the propellant, the flame jet length is also increased by the microwave. In the case of the 1500 W jet length as the benchmark, for every 250 W increase in the microwave power, the corresponding increase in the flame jet length is close to 20%, indicating that the microwave enhancement effect of the energy on the flame length does not appear to be attenuated. In general, while keeping the gas working medium and propellant flow unchanged, the input of the microwave has a relatively obvious improvement on the flame length. In the experiment, the gas flow rate was kept constant by the airflow meter, the microwave power was gradually increased, and the temperature at the stable time of the plasma jet was measured with the increase of microwave power.

**Figure 9:**
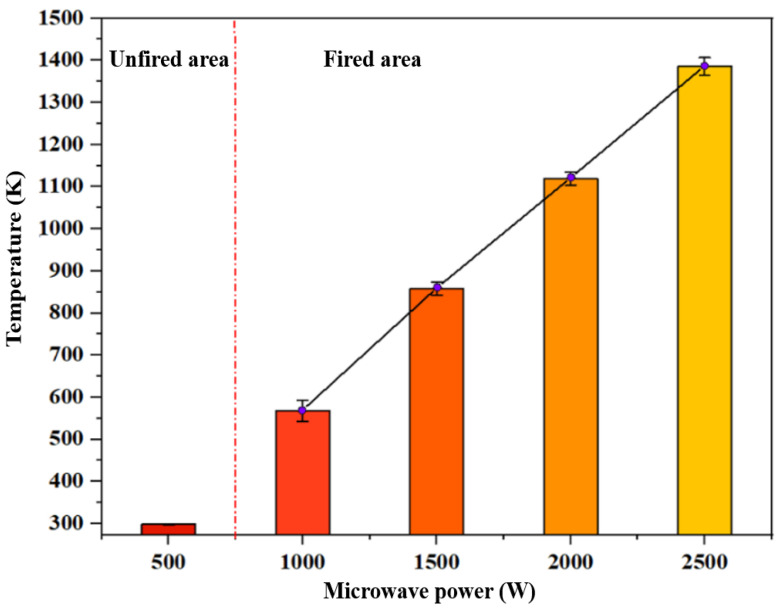
Plasma temperature corresponding to different microwave power under stable conditions.

**Figure 10:**
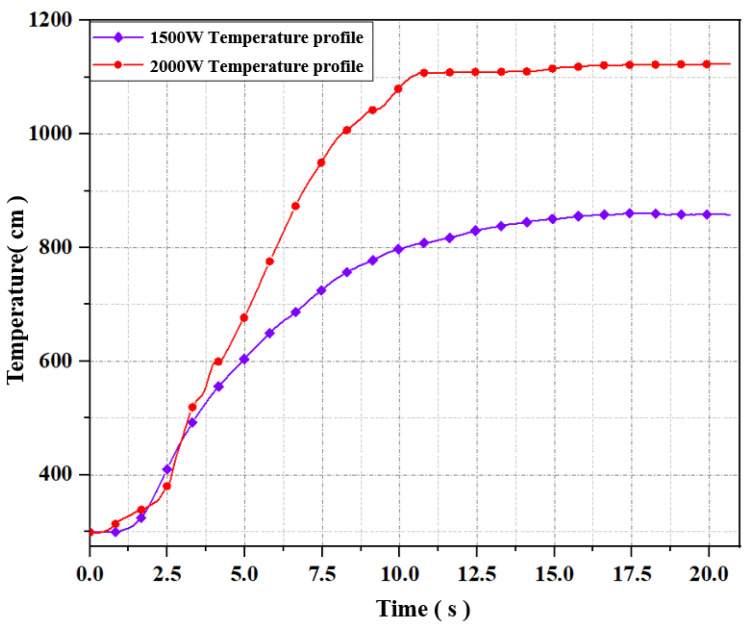
Heating curves under two microwave powers.

Figure 9 shows the plasma temperature corresponding to different microwave power during stable combustion, where the error bar represents the upper and lower fluctuation range of the flame temperature during the stable combustion period. Since there is no microwave plasma generation in the case of 500 W, it can be regarded as the room temperature during the experiment, which is 298.33 K. In the case of 1000 W, the plasma jet is short, and the collected data is the temperature of the hot air flowing upward in the tube after the plasma jet is generated or the invisible outer edge of the flame, so the temperature fluctuation range is relatively large and relatively inaccurate. When the microwave power is above 1500 W, the thermocouple can directly measure the plasma jet itself. Taking the temperature of 1500 W as the benchmark, the temperature rise ratio corresponding to the three cases of 1500 W, 2000 W, and 2500 W is approximately 45%. Overall, with the increase in microwave power, the temperature of the plasma jet showed an upward trend. When the microwave power was 2500 W, the temperature of the plasma jet reached 1400.15 K. Figure 10 is the heating curve under the microwave power of 1500 W and 2000 W. It can be seen from the figure that the increase in microwave power not only increases the temperature at a stable rate but also affects the temperature rise rate. The temperature rise rate is faster under the microwave power of 2000 W than that under 1500 W. Under the continuous action of a 2000 W microwave, the time required for the combustion temperature of the propellant to rise to 800 K is 5.75 s, while the time required for 1500 W is about 10 s, which means that the increase in power also has a certain enhancement effect on the temperature rise rate.

### 3.2. Influence of Gas Flow Rate on the Combustion Characteristics of Plasma Torch

#### 3.2.1. Influence of Gas Flow on Air Plasma Jet

Before the ADN-based liquid propellant is sprayed into the resonant cavity, the microwave device generates a strong electric field in the resonant cavity through microwave energy to ionize the gas to form air plasma. When the gas flows in the cylindrical quartz tube, different gas flow rates will make the gas motion state different.

The ratio of the inertial force and the viscous force of the fluid itself is defined as the Reynolds number. The Reynolds number is usually used to reflect the flow state of the fluid. It is a dimensionless number and can be used to judge the state of different gases and liquids. The Reynolds number is represented by Re, and its formula is as follows:

(8) Re=ρvl/η

**Figure 11:**
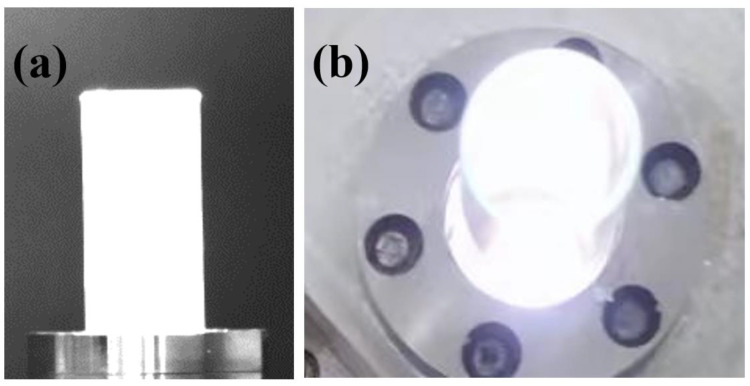
Plasma generation under 2 L/min gas flow rate. (**a**) Plasma generation on quartz glass surface; (**b**) Plasma generation inside the resonant cavity.

In this paper, the gas flows into the quartz tube from the lower end, and the quartz tube can be regarded as a circular tube channel with a certain diameter. For the Reynolds number of a straight pipe, the critical value of the Reynolds number is between 2000 and 2300. If it is less than this value, it can be regarded as laminar flow, and if it is greater than 2300, it can be regarded as turbulent flow. If the Reynolds number is 2300 as the critical value, the corresponding critical air velocity is 38 L/min. In the experiment, keeping the flow rate of the gas working medium below 33 L/min, it can be considered that the gas working medium is kept in a laminar flow state. In the study of the effect of the gas flow gas working medium on the plasma jet, the microwave power was kept at 1500 W, and the gas working medium flow rates were set at 2 L/min, 8 L/min, 14 L/min, 20 L/min, 26 L/min, and 32 L/min, respectively. The influence of gas working medium flow on plasma generation and development is shown in Figure 11. When the airflow rate is maintained at 2 L/min, the breakdown of the gas working medium can be achieved under the action of microwaves, but no plasma jet is generated; the experiment of 2 L/min shows that the gas working medium flow rate is higher than in small cases, although the device can ionize the gaseous working medium, it cannot produce a significant plasma jet.

**Figure 12:**
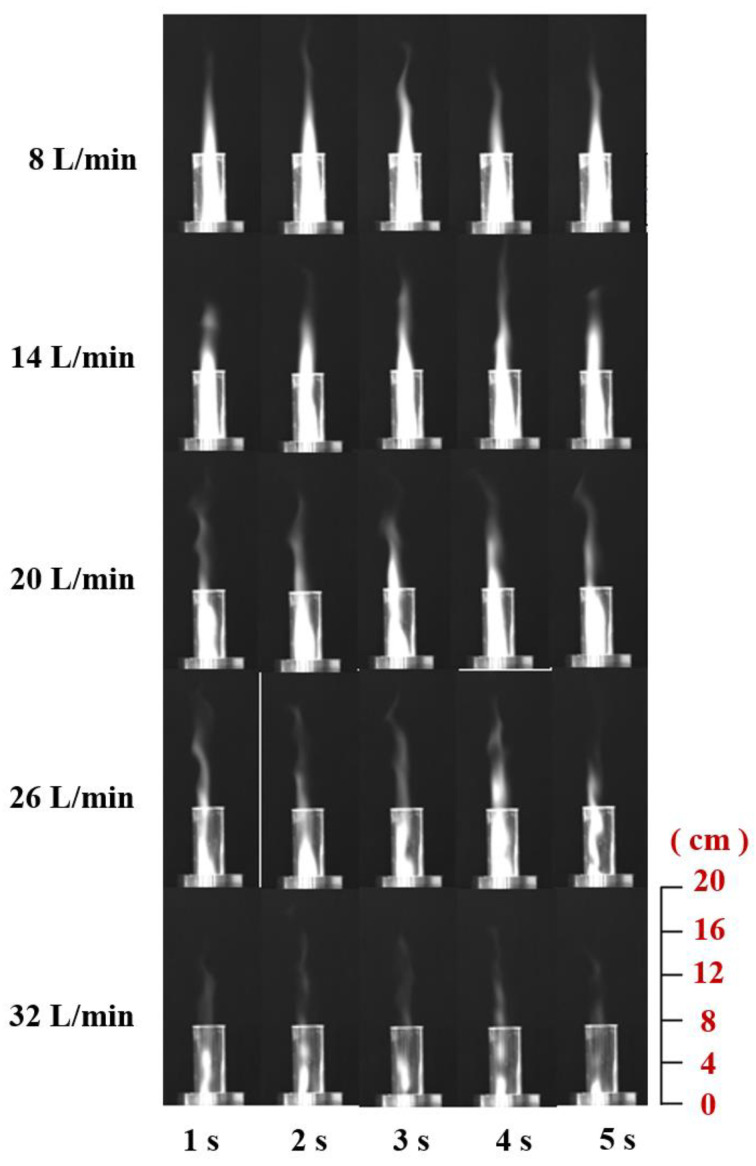
Plasma jets under different gas working flow rates.

As shown in Figure 12, when the gas flow rate is 8 L/min and 14 L/min, the gas can generate a relatively stable and continuous plasma jet under the action of microwaves. The plasma jet gradually weakens and becomes thinner from bottom to top, and the color gradually becomes lighter. The shape and area of the plasma jet at different times are not very different. When the gas flow rate increased to 20 L/min, the length of the plasma jet was significantly shortened compared to 14 L/min, and the width of the jet was also reduced. Continuing to increase the flow rate of the gas working medium, the plasma jet is ejected along with the rising gas flow without being ionized because the gas flow rate is too fast. This situation reduces the area of the plasma jet and greatly reduces the continuity of the plasma jet. In the case of 26 L/min, some of the plasma jets have been interrupted. The jet area is significantly reduced, and the end blur is serious; when the gas working fluid is 32 L/min, although the plasma can still be generated, the high-speed gas flow rate makes the plasma jet very weak and it can no longer be stable and continuous. During the experiment, when the gas flow rate is too large, the sound of the airflow flowing out of the quartz tube can be clearly heard.

**Figure 13:**
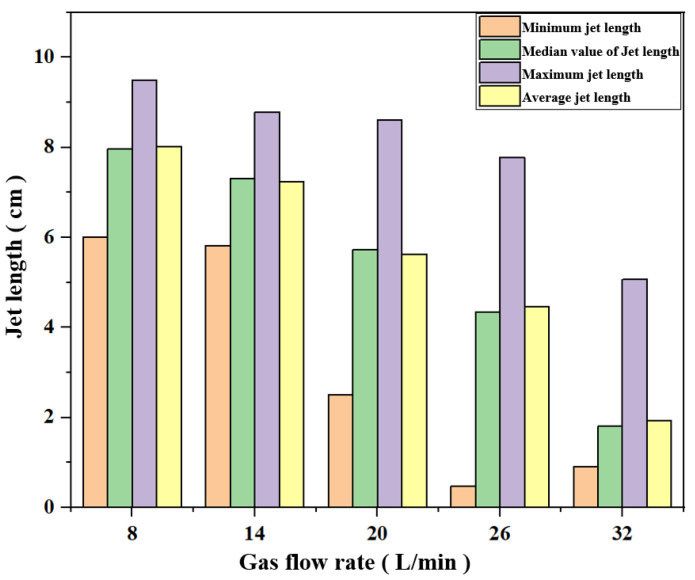
Median, average, and maximum values of plasma jet under the action of different gas flow rates.

Figure 13 shows the results of the air plasma jet length under the action of different gas working medium flow rates. It can be seen from the figure that when the gas flow rate is 8 L/min and 14 L/min, the fluctuation range of the jet length is small, and the median value of the longest jet, the shortest jet, and the jet length is not much different (the median difference is 6 mm). When the gas flow rate is greater than 14 L/min, the reduction of the air plasma jet length is more obvious. Taking the jet length of 14 L/min as the benchmark, the reductions in the jet length of the subsequent three groups were 21.6%, 40.4%, and 75.3%, respectively. The minimum value of jet length also has a big gap after 14 L/min and the longest value of jet. In general, when the gas working medium flow rate is 8 and 14L/min, the length of the plasma jet is relatively stable; and when the gas working medium flow rate is above 14 L/min, the length of the plasma jet continues to shorten, and all the jet length statistics are in a downward trend.

**Figure 14:**
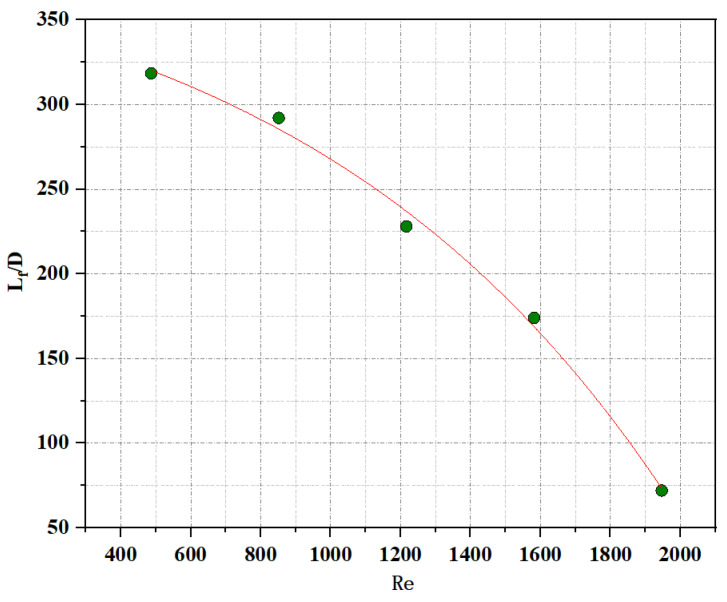
Dimensionless treatment of air plasma jet length.

Figure 14 shows the dimensionless processing of the plasma jet length under different gas working medium flow rates. The abscissa is the Reynolds number, and the ordinate is the median value of the air plasma jet length ratio to the inner diameter of the quartz tube to achieve dimensionless parameters. Through the parameter fitting after dimensionless, it can be found that the data is distributed in exponential form, and the fitting line can be expressed as $\frac{Lf}{D}=-54.88e\left(\frac{\frac{Pmw}{Pmin}}{1079.60}\right)+406.32\left(R^{2}=0.99\right)$.

To sum up, the gas flow rate is closely related to the generation of the plasma jet, and the gas flow rate is too large or too small to have an adverse effect on the length of the plasma jet. When the gas flow rate is too small (2 L/min), although the gas working medium is relatively completely ionized, it is difficult to generate a long jet; and when the gas working medium flow rate is large, the plasma jet will also be affected, and a large amount of gas will not be ionized. Exiting the reaction zone with the upward gas flow rate results in a shortening of the plasma jet to the point where continuous plasma generation is not possible. Subsequent attempts were made to continue to increase the gas working medium flow. The experiment proved that a larger flow of gas working medium would have a great impact on the generation and maintenance of plasma, and plasma maintenance could not be achieved under the condition of 38 L/min.

#### 3.2.2. Influence of Gas Flow Rate on the Flame of ADN-based Liquid Propellants

**Figure 15:**
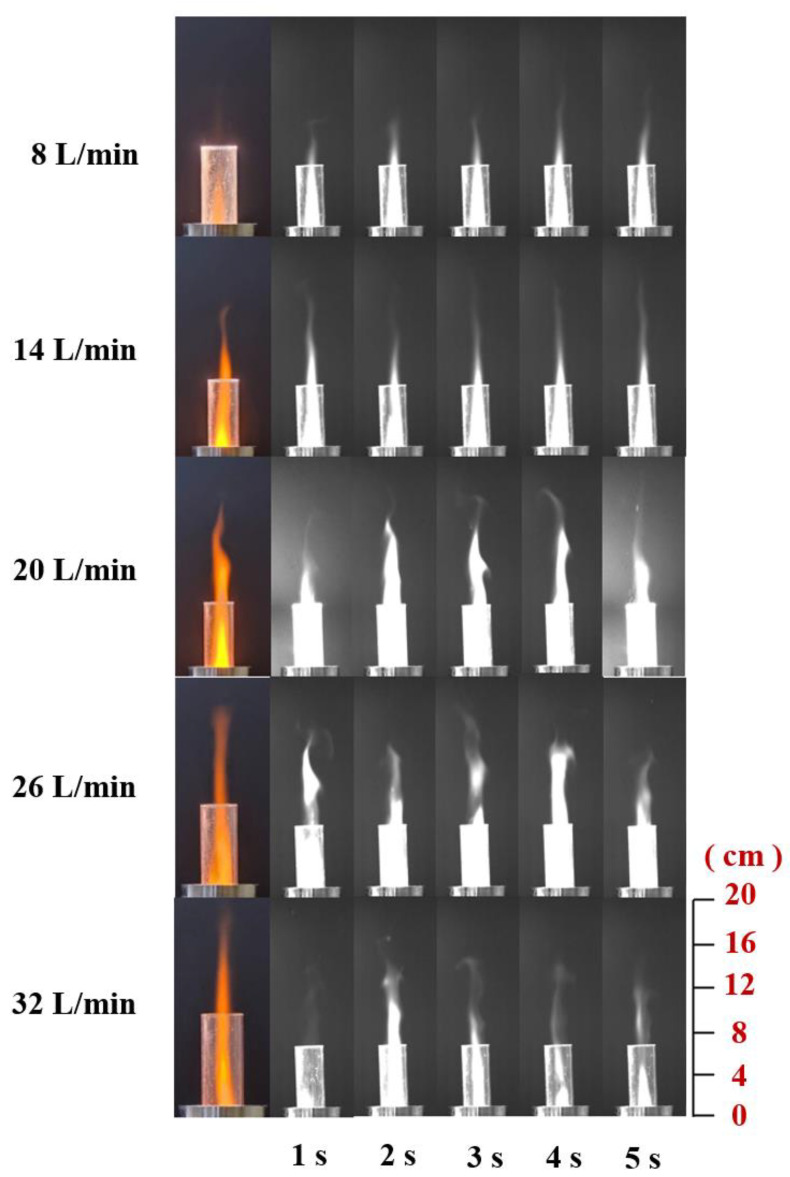
Combustion of ADN-based propellant at different gas flow rates.

It can be seen from Figure 15 that the color of the flame burning brightness, as well as the height of the inner flame, rises first and then decreases and that the high flow rate of the gas working mass will lead to the decrease of the flame brightness and the reduction of the jet area, and the color of the ADN-based liquid propellant burning under the action of the high flow rate of the gas working mass is light. From the images at different moments (black and white images), it can be seen that when the gas work flow is small, the combustion reaction is relatively not intense, the brightness in the quartz tube is low, and the length of the flame jet formed is short, but the length and area of the flame jet are more stable; while the gas work flow is at 20 L/min, the flame jet length of combustion has a more obvious increase, and the brightness in the quartz tube is extremely high, which means that the combustion reaction is more intense in this case; when the gas flow rate is 26 L/min, the flame jet generated by combustion begins to show a discontinuous phenomenon, and the high-speed airflow blows part of the flame away from the main body of the jet, and the jet height decreases compared with 20 L/min. When the gas flow rate is 32 L/min, the brightness in the quartz tube decreases significantly compared to 20L /min, and the flame jet height also decreases significantly, but the combustion reaction can still be maintained at a gas flow rate of 32 L/min.

**Figure 16:**
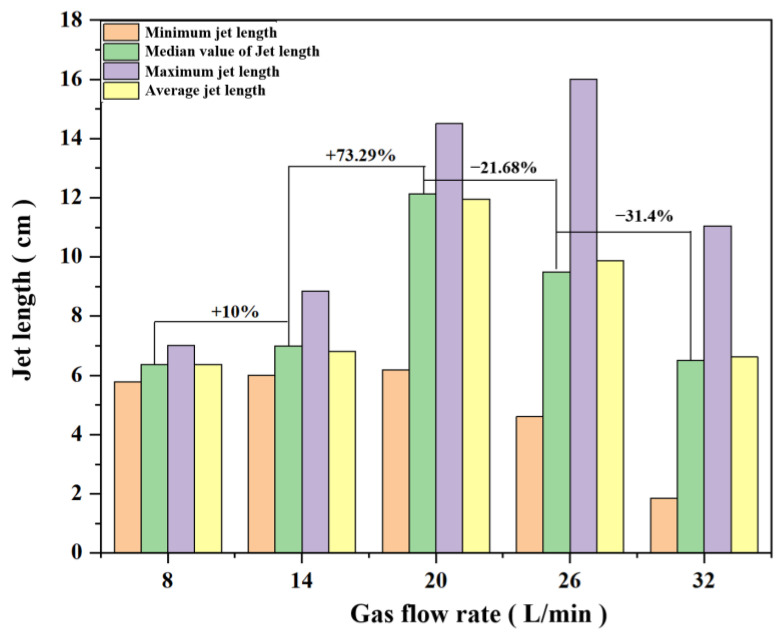
Median, average, and maximum values of propellant flame length under the action of different gas flow rates.

Figure 16 shows the statistical parameters of the propellant combustion jet at different gas flow rates. As can be seen from Figure 15, when the gas work-quality flow rate is below 26 L/min, it has little effect on the minimum value of the combustion jet, and the minimum values of the first three groups of experiments are all around 6 cm. When the gas flow rate is 8 L/min, the gas flow rate is low, and the maximum value of the combustion jet flame produced is not much different, and the flame jet length is relatively stable, but the jet length is short, and the maximum value of the jet length is 7.02 cm; when the gas flow rate increases to 20 L/min, the combustion jet shows an obvious growth, and its maximum value is 14.51 cm, which is nearly double compared with 14 L/min. At the same time, the median value of the jet also showed a significant increase of 73% compared to 14 L/min, indicating that the flame combustion was better at 20 L/min. The further increase in the gas flow rate led to a decrease in the average length of the jet. At the gas flow rate of 26 L/min, the maximum value of the flame jet increased, but the median value of the jet length decreased more significantly with the average and minimum values, in which the median value decreased by 21.68% compared with 20 L/min. The maximum value of the jet length at 32 L/min also decreases significantly, by 5 cm compared to 26 L/min; the median value decreases by 31.9% compared to the median value at 26 L/min, totaling 3.06 cm. proceeds, resulting in a shortening of the flame length.

**Figure 17:**
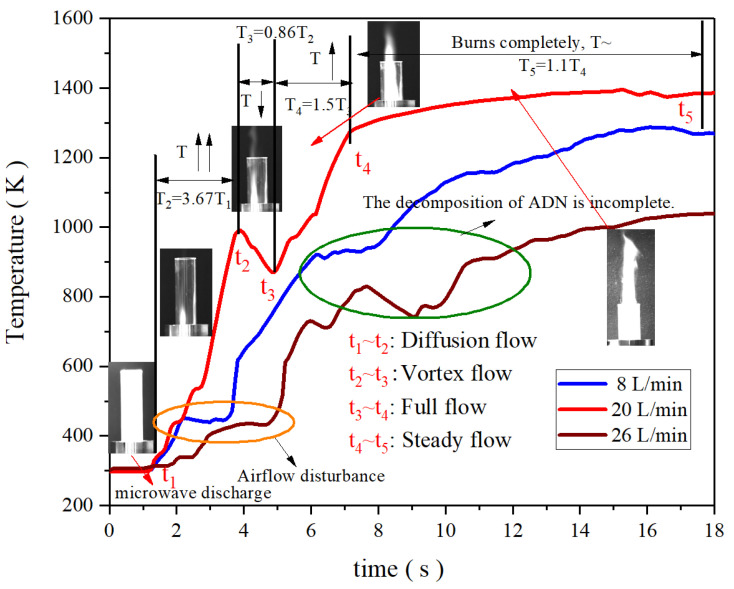
Temperature rise curves of ADN-based liquid propellants with different gas flow rates.

Figure 17 shows the temperature rise curves of ADN-based liquid propellants at 8 L/min, 20 L/min, and 26 L/min of gas flow rates. Taking temperature as an indicator, the temperature change process is divided into four modes according to the way the gas flows, including diffusion flow, vortex flow, full flow, and steady flow. When microwave discharge begins, air diffuses and flows in the resonator and the temperature rises rapidly to peak T_2_. When the spray and the airflow are mixed, a vortex occurs, ADN cannot be completely decomposed, and the temperature is briefly reduced to T_3_. With the increase of airflow rate, the oxygen content in the resonator increases, the oxidation reaction of methanol occurs, and the flame temperature rises sharply. Finally, the propellant burns more completely as the gas flow stabilizes. In the case of 8 L /min, the temperature rise curve shows a stepped rise, with a flat period of nearly 1 s after the temperature climbs, after which the temperature continues to rise until it reaches the maximum temperature. The temperature rise curve for 20 L/min also has a stepped rise, but the duration is very short and distributed within 2.5 s at the beginning of the rise. Unlike the 8 L/min case, the temperature rise curve for the 20 L/min case has a period of decline, a brief drop in temperature followed by a rapid rebound and peak temperature. In contrast, the step-like distribution of the temperature rise curve is more obvious and longer in duration for the case of 26 L/min of gas flow rate. The curve also has a short drop in temperature after reaching the extreme value, but the duration is significantly longer than that of 20 L/min. The temperature extremes in the heating curve and a brief drop may be due to the decomposition of the ADN itself, before reaching the first extremes of the ADN itself in the microwave action exothermic, and in the case of 8 L/min there is no significant temperature drop, but a period of flat, which may be due to the lower gas flow rate. In the case of the same input power, the ADN-based liquid propellant absorbed more power so that the combustion temperature does not appear to drop significantly after reaching the extremes due to decomposition. The comparison of the temperature profiles shows that the temperature rises faster at 20 L/min and the duration of the temperature steps during the warming process is shorter.

## 4. Conclusions

In this paper, through the experiment on the actual effect of the resonator under different microwave power and gas flow rates, the following conclusions are drawn:

(1) Microwave power plays a leading role in the ignition performance of microwave plasma and the combustion effect of propellant. When the gas flow rate is constant, the increase of microwave power can effectively increase the length and temperature of the air plasma jet. In the case of stable propellant combustion, stopping the microwave power input leads to a cessation of the combustion reaction.
(2) After achieving combustion of ADN-based liquid propellants, the effect of gas flow rate on the flame jet and temperature both increase and then decrease, with the highest flame jet length and temperature at 20 L/min.
(3) The changes in microwave power and gas flow rate did not change the wavelength range corresponding to free radicals in the spectrum but had a certain influence on the spectral intensity of free radicals.

## Figures and Tables

**Table 1:** Uncertainty of parameters.

Parameter	Uncertainty
Pipe diameter	3.57%
Tube length	3.3%
Temperature	5.55%
Gas flow rate	4%
Propellant flow	1%

## Data Availability

Not applicable.
